# ‘The god of criminals is their belly’: diet, prisoner health, and prison medical officers in mid-nineteenth-century English and Irish prisons

**DOI:** 10.1017/mdh.2023.36

**Published:** 2024-07

**Authors:** Catherine Cox, Hilary Marland

**Affiliations:** 1 University College Dublin; 2 University of Warwick

**Keywords:** prison diet, prison medical officers, discipline, health, experimentation

## Abstract

Existing scholarship on prison diets has emphasised the role of food and its restriction as a key aspect of the deterrent system of prison discipline introduced in the 1860s. Here we suggest that a strong emphasis was placed on dietary regulation after the establishment of the reformist, but also ‘testing’, separate system of confinement in the mid-nineteenth century. While the impact of diet on the physical health of prisoners was a major concern, we argue that the psychological impact of food was also stressed, and some prison administrators and doctors argued that diet had an important protective function in preserving inmates’ mental wellbeing. Drawing on a wide range of prison archives and official reports, this article explores the crucial role of prison medical officers in England and Ireland in implementing prison dietaries. It highlights the importance and high level of individual adaptations to dietary scales laid down centrally, as a means of utilising diet as a tool of discipline or as an intervention to improve prisoners’ health. It examines the forays of some prison doctors into dietary experiments, as they investigated the impact of different dietaries or made more quotidian adjustments to food intake, based on local conditions and food supplies. The article concludes that, despite central policies geared to establishing uniformity and interest in new scientific discourses on nutrition, a wide range of practices were pursued in individual prisons, mostly shaped by practical rather than scientific factors, with many prison medical officers asserting their autonomy in making dietary adjustments.

## Introduction

From the establishment of the modern prison system in England and Ireland in the mid-nineteenth century, diet formed a cornerstone of prison discipline and a key aspect of the maintenance of prisoners’ health. To date, the scholarship on prison diet has largely focused on the imposition of more deterrent-based approaches after the 1860s, when penal regimes increasingly centred on ‘hard labour, hard board, hard fare’, with diet kept ‘as minimal as possible without impairing health’.^1^ This article examines how prison diet became a crucial consideration in managing prisoners’ health, as the separate system of confinement was rolled out across the British Isles after the 1840s. During this formative period, the management of prison diet became a vast food experiment and the subject of extensive investigation and inquiries. As this article highlights, prison medical officers performed a crucial role in moderating diet, which set up tensions between their conflicting duties of protecting prisoners’ health and enforcing prison discipline. From the early years of the separate system, experiments with prison diet were critical to the success of the new system of discipline and, while acknowledging that the boundary between mental and physical health was shaky, our article highlights the emphasis that was placed on the relationship between psychological wellbeing and diet. This was especially important under the separate system that emphasised the role of the mind in supporting prisoners’ reform.

In assuming the right to punish prisoners, the state, as James Vernon has noted, ‘assumed an obligation for their welfare, if only to maintain them in a position of “bare life”, so that punishment was still possible’.^2^ As such, the role of the prison medical officer was vital in both convict and local prisons. In the former, government prisoners served a probationary period in separate confinement prior to transportation. After the decline in transportation, and the modification of disciplinary regimes in the 1850s and 1860s in England and Ireland, prisoners were moved to public works prisons to complete their sentences, where they were put to hard labour in association with other prisoners. Local prisons served a variety of functions, chiefly to detain prisoners awaiting trial, debtors, prisoners condemned to capital punishment, and those sentenced for terms of up to two years.^3^ The management of prison diet in convict and local prisons in both countries was subject to compliance with the various prison dietaries laid down centrally from the early nineteenth century and supplementary instructions generated by numerous official reports.^4^ However, as this article demonstrates, these were adapted by prison medical officers in many individual prisons. Prison doctors were also required to check on the preparation of food and quality of the ingredients. Along with assessing prisoners’ fitness to undergo punishment, hard labour, or placement on punishment diets, doctors made decisions about whether prisoners’ diets needed to be enhanced on health grounds, either by admitting them to the prison infirmary or granting extra items, and, in situations where prisoners were declared insane, their removal to an asylum would give prisoners access to a more generous and appealing diet.^5^ Such decisions had cost implications as food was a significant expenditure for prisons throughout the mid- to late nineteenth century, and cost acted as a strong stimulus or potential brake to prison doctors’ interventions on diet. Local magistrates, responsible for managing local prisons, sought economies in dietaries when faced with the disgruntled ratepayers who funded them,^6^ while pressure from central prison authorities to significantly reduce costs became more pronounced. The latter became critical in the 1860s as the expanding and costly prison estates in both countries were disparaged by critics of the reformative impulses behind the separate system seeking a more punitive and cost-efficient prison system.^7^

Even with cost restraints and the requirement placed upon them to uphold prison discipline by means of dietary restrictions, prison doctors demonstrated their ability to use dietary regulation to manage and even improve prisoners’ health. At the same time, they sought to raise their status within the prison service and the medical profession as a whole by demonstrating their expertise in the expanding field of prison medicine, which demanded, they argued, unique skills in assessing and managing mental illness, including the detection of feigning, adjudicating prisoners’ fitness to undergo the rigours of the prison regimes and hard labour, and managing dietaries.^8^ For prison doctors, the balance between supporting penal discipline and the demands of maintaining the health of their prisoner-patients, the twin and conflicting pillars of their roles, was never more significant than in the case of food, given its overwhelming importance not only for prisoners’ physical health but also their mental state.

In historical scholarship, prison medical officers have stood accused of an eagerness to impose prison discipline, including adjustments to prison diet, to the detriment of prisoners’ health. However, Ciara Breathnach has argued that in the late nineteenth century Irish convicts were able to assert agency to obtain more food and exercise or less work.^9^ Joe Sim has contended that the constraints of ‘dual loyalty’ in general hampered prison medical officers’ ability to work either independently or benevolently. Moreover, Ian Miller has described the intense public and expert debates around the tensions between sustaining prisoner health while ‘serving meals of a punitive nature’, against a backdrop of the emergence of food science and prison-based research into nutrition and a much wider interest in institutional diets, notably in workhouses.^10^ The principle of ‘less eligibility’ and widely-aired fears that prisoners might be fed better than poor working people at liberty added to this tension.^11^ This article advances an already rich scholarship on prison medical officers and dual loyalty, drawing on detailed examples to explore prison doctors’ agency and capacity to act in varied ways to moderate prison diet in individual prison contexts, with some prisons enabling them to exert their authority more extensively than others. Prison doctors embodied very different attitudes towards their work; while some were supportive of the disciplinary regimes in place in prisons, others sought to improve the health of their charges or at least mitigate the worst aspects of prison discipline.

A number of prison doctors also took the opportunity to experiment with prison dietaries in their own prisons, including the imposition of testing dietary regimes on prisoners in the interest of establishing where the minimum requirement for the maintenance of health (generally measured by prisoners’ weight) might lie, abetting the imposition of prison discipline and superimposing their own versions of dietary management on prison regimes.^12^ Here we explore a selection of these experiments, including those imposed following the introduction of the separate system in Pentonville in London in 1842 and Mountjoy in Dublin in 1850. While a handful of prison doctors, including William Milner at Wakefield, conducted a series of complex, long-term experiments, others ‘experimented’ in a more quotidian fashion as they moderated and supplemented the diets of individual prisoners or adjusted dietaries within the prison as a whole, adapting to local conditions and practices.

Our focus here lies not with the use of food as an instrument of power in prison in the form of food refusal and hunger strikes or with the scientific food discourses of the mid-nineteenth century, which have been extensively analysed by other scholars.^13^ Rather, our main objective is to investigate the ways in which prison medical officers took on board a broad range of factors in considering prison diets, focusing on the physical and mental health and ill health of inmates and their ability to fulfil the tasks of reformation in the early years of the separate system, as well as hard labour in the latter part of the century. We assess how diet became a disciplinary device or a tool that enabled prison medical officers to maintain or improve health in individual prisons. Through an exploration of individual prison medical officers’ experiments and findings on diet, which largely judged the value of dietary initiatives based on prisoners’ weight loss and gain, our article also questions the assertion of Price and Godfrey, based on their measurement of prisoners’ BMI at committal and release, that ‘few convicts lost considerable weight in prison’.^14^

In both countries a great deal of attention was directed towards prison dietary, and prison administrators and doctors actively compared and critiqued dietary provisions in English and Irish prisons. There appears to have been more interest in dietary experiments in the English context, though Ireland also tested new dietary initiatives, such as a meatless regime, in the early years of separate confinement. After 1845 Irish prison diets were also strongly shaped by limited food supplies during the Great Famine. While England did not experience the extremes of famine conditions in the mid-nineteenth century, studies of the adequacy of food supply and the affordability of food for the labouring population highlighted the insufficient nutritional standards of many of the nation’s poor.^15^

## Diet and the Pentonville Experiment

As experiments with the new disciplinary system of separate confinement were carried out, at Millbank Penitentiary after 1816 and Pentonville Model Prison after 1842, and as it was introduced across the expanding prison estates in England and Ireland during the mid-nineteenth century, diet was acknowledged to be an important device in fine-tuning prison regimes, but also one that might imperil prisoners’ health. The dire consequences of dietary adjustments had been recognised during the 1820s when potatoes and most meat were removed from the diet at Millbank Penitentiary, where nearly half of the 860 inmates developed symptoms of scurvy and thirty-one prisoners died.^16^ The subsequent inquiry alluded to the pressures that had prompted the reduction in diet. Millbank had been described as a ‘fattening house’, and the principle of less eligibility invoked, ‘that honest labourers out of doors had not so good food as the prisoners found within the walls of the prison’.^17^ Though the findings of the final report referred chiefly to physical health, disease, and the deaths at Millbank rather than mental wellbeing, it was observed that ‘depression of spirits’ induced by solitary confinement had acted as a ‘moral cause’ of the sickness, making the prisoners susceptible to disease.^18^

Pentonville Model Prison in London, the first specially designed prison to adopt the new regime of separate confinement when it opened in 1842, subjected its prisoners to extensive dietary experimentation, highlighting the absence of ethical considerations when experimenting on convicts without their consent.^19^ The disciplinary regime at Pentonville Prison was itself referred to as an ‘experiment’ that would push the limits of separate confinement in its efforts to stimulate deep-seated reflection and reform amongst its inmates. Applying a ‘testing’ environment for its carefully selected prisoners, men aged between eighteen and thirty-five and in good health were exposed to eighteen months of extreme cellular isolation. Communication with other prisoners was forbidden, and the men ate, worked, and slept in their cells, where they were confined for twenty-three hours of the day. When removed from their cells to attend chapel and to exercise, the convicts were hooded to prohibit recognition by other prisoners.^20^ As part of the meticulous design of Pentonville Prison for separate confinement, Surveyor-General of Prisons, Joshua Jebb, who would subsequently oversee the design of Mountjoy Model Prison in Dublin, had carefully considered the architectural and mechanical aspects of prison diet, including the weighing and distribution of food around the prison three times a day. Pentonville’s Assistant Chaplain John Burt would later reflect that Pentonville’s Commissioners had ‘bestowed much attention upon the subject of diet’ in the first year of operations.^21^ During the first year, a series of five diets was tested, with inmates being regularly weighed, to assess the ‘scientific minimum’ of the diet, without it being hazardous to health.

Following inquiries into the dietary of convict hulks, Poor Law Unions and hospitals, and taking the advice of Pentonville’s two Medical Commissioners, Sir Benjamin Brodie and Dr Robert Ferguson, Pentonville’s Medical Officer, Dr Owen Rees, initially proposed the adoption of the No. 3 diet (see Table 1 below), which he regarded as suitable for prisoners in separate confinement and not undergoing hard labour.^22^ However, against his advice, it was the prison’s most restrictive diet, the meagre No. 1 diet, that was adopted in 1843, with the proviso that it would be increased if necessary. This resulted in weight loss amongst many of the convicts, who described how they felt ‘faint & sinking’, through lack of food: ‘they wished to have more bread’.^23^ Of the fifty prisoners placed on the No. 1 diet, 62 per cent lost an average of five pounds in a month.^24^ After a three-month trial, it was concluded that it was ‘inadequate to the preservation of health – the loss being considerable, and accompanied by weakness’.^25^ An increased quantity of bread was introduced in April 1843, nudging the diet into the No. 2 category, and, while this appeared to prevent further weight loss, there were many complaints of lassitude and debility amongst the prisoners, and the diet was subsequently enhanced to No. 3 with additional meat. Use of this diet continued for six months, but the prisoners once again lost weight, and in November the No. 4 diet was introduced, with the addition of half a pound of potatoes at dinnertime. Even then weakness was observed in prisoners, who complained of a sensation of ‘sinking’. Finally, a quarter pound of bread was added to the breakfast allowance, introducing the No. 5 diet. One year after initiating the No. 1 diet, it was reported that ‘the amount and kind of diet necessary and sufficient to sustain the health and strength of the prisoners had been achieved’, and Dr Rees expressed satisfaction with its effects.^26^ Though many prisoners continued to lose weight, this was attributed to the positive effects of the prison discipline and the convicts’ improved bodily condition.^27^

**Table 1.**
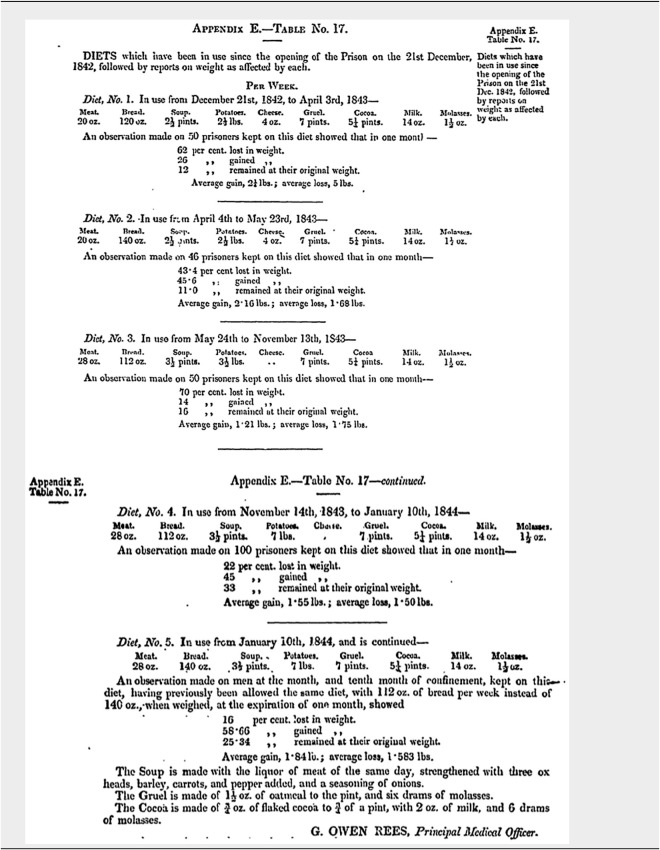
Pentonville Prison Diets

Second Report of the Commissioners for the Government of the Pentonville Prison, 1844 [536], 53–4.

Even before Pentonville took in its first convicts, opponents of the separate system highlighted the potential damage the prison’s extreme form of separate confinement would inflict on prisoners’ health, particularly their mental wellbeing.^28^ The cruelties of dietary limitation had already been pointed out in 1837, when surgeon J.G. Malcolmson described the impact of severe dietary restriction under solitary confinement, in this case in military prisons in India, which produced ‘intractable forms of disease’, ‘ruinous to body and mind’.^29^ By 1843, as the new dietary scales were tested in Pentonville, symptoms of mental breakdown were widely reported in the prison, with cases of depression, insanity, mania, hallucinations, and ‘faintings’ disrupting the regime and its objectives. The long process of reaching a satisfactory diet and the fact that Rees’ advice was initially overruled also highlighted the limits of the prison medical officer’s authority, during a period when diet was being identified as a factor that might provoke or potentially protect against mental breakdown as well as physical debilitation and disease.

Concerns about prison diet appear to have been informed by the principles of ‘constitutional medicine’, which explained illness in terms of living conditions and personal histories, recognising the interdependence of mental and physical factors in producing illness and disease, and seeing conditions such as irritability of the nerves, fretfulness, fatigue, and depression as related to the somatic state of debility.^30^ Constitutional medicine also acknowledged the relationship of food deprivation with other ‘depressing conditions’, so much so that the term ‘starvation’ was believed to refer not just to want of food, but to other forms of privation. As such, terms such as fainting, sinking, debility, and weakness, terminology widely shared by prisoners and medical officers, acknowledged the close connections between mental distress and physical decline and debility, which resulted from a combination of enforced solitude and an extremely limited diet. Expressing deep-seated reservations about the likely success of the separate system, Pentonville’s Medical Commissioners, Brodie, who ascribed to the ideas of constitutional medicine, and Ferguson, pointed out ‘there are few minds which would not suffer from the monotony and *ennui* of this mode of existence’ even during shorter periods than eighteen months.^31^

By 1847 the Pentonville Commissioners were convinced that the prison’s diet was satisfactory; though weight was regarded as an ‘imperfect’ test, ‘it nevertheless affords a strong presumption as to the health of the body; and considering how deeply the frame is always affected when the mind is depressed, it is an evidence also that the moral discipline of the prison has not acted injuriously on its inmates’.^32^ A year later, Jebb asserted that the push to establish an appropriate dietary was largely to ascertain ‘the precise quantity required to support the Prisoners against the depressing influences of separation’.^33^ Nonetheless, high incidences of mental distress continued to be reported for the rest of the decade in Pentonville, despite various mitigations to the regime, notably a reduction in the length of separate confinement to twelve months by 1848. Rather than being associated with mental and moral reform, Pentonville stood accused of provoking high rates of mental distress and destroying the minds of the men confined there.

Reflecting back on the separate system at Pentonville in 1852, John Burt, one of its strongest advocates, pointed out that while the ‘liberal dietary’ finally adopted was objected to by those who saw it as ‘an undue indulgence to the criminal’, if criminals were to be severely punished, they must be ‘sustained’:

When heavy inflictions are laid upon them, and the support which the body requires is withheld, their health will in many cases be impaired, and the consequences will be as costly as the treatment would be inhumane. The greater grief induced by a discipline which is effective may *possibly* require increased nutrition.^34^

While largely positive about the impact of the separate cell, which had a ‘very corrective effect upon the mind of a prisoner’, Jebb would again underline the adverse effects of an insufficiency of diet on the mental and physical condition of Pentonville prisoners in separate confinement in his evidence to the Carnarvon Committee twenty years later in 1863.^35^ He re-asserted the importance of a ‘good diet’, reflecting that it counteracted the ‘depressing influences of separate confinement’, concluding that men undergoing separate confinement might need more food than those undertaking hard labour.^36^

## Dietary experiments and the separate system at Wakefield Prison

In 1843 a new set of prison dietaries for local and convict prisons was set out by Home Secretary Sir James Graham for each class of prisoner, including those sentenced to hard labour, intended to be sufficient to maintain health and strength. However, as these were recommended and not mandated dietaries, they were adopted in only a minority of prisons, whereas many prison medical officers adapted diets in response to local conditions, cost, and their impact on the prisoners’ health. Dr William Milner, Medical Officer to the Convict Department of Wakefield Prison, carried out several experiments with prison diet between the late 1840s and 1860s. He was also alert to the impact of the separate system on prisoners’ health and in 1847 expressed concern about the ‘unmanageable’ delusions experienced by prisoners in separate confinement, which he related to their limited diet. His response was to increase dietary allowances along with periods of exercise, concluding that convicts benefitted from these modifications, thereby ‘shewing that the system of total separation was not universally applicable’.^37^

However, despite Milner’s interventions, in 1849 Joshua Jebb raised a serious complaint about the state of health of a group of convicts who were being transferred from Wakefield’s Convict Department to Portland Prison, having completed their probationary period of separate confinement in Wakefield. Jebb described the men as being in a ‘very low condition’ and ‘altogether unfit’ for employment at hard labour.^38^ Some had to be detained in London en route, as they were considered too ill to travel onwards to Portland. One was found to be ‘insane but quiet and harmless, another in an advanced state of consumption, 7 others with scorbutic swellings and a large number with spongy gums’.^39^ Jebb concluded that their low state of health was attributable to either insufficient diet, the long period of separate confinement that they had undergone (some had been held for six months at Millbank and then a further twelve at Wakefield, greatly exceeding the maximum of twelve months by this time), or most likely a combination of these causes.^40^

Several of the prisoners transferred to Portland had complained bitterly about the impact of the reduced diet at Wakefield, which, as Jebb confirmed, was intended first and foremost to produce savings. Alterations to the rations, which were supposed to be the same as those finally adopted at Pentonville, had been recommended by Frederic Hill, who became Inspector of Prisons for the north of England and Wales in 1847. Hill claimed that the health of the prisoners had improved following the change of diet and that they had gained weight, at the same time saving over £1,000 a year.^41^ Jebb disputed this, asserting that the question of diet should not ‘be regarded merely as one of pounds shillings and pence:- the question is how much is necessary to enable them to bear the discipline without greater depression to their physical and mental powers’.^42^ He also warned of the risks of so enfeebling convicts that they would be unfit for transportation and end up languishing in an invalid establishment at the state’s cost. Following Jebb’s complaint, between March and May 1849 Wakefield Prison experienced an outbreak of cholera, which coincided with weight loss among the convicts. At this point Milner concluded that this was due to ‘the want of more animal food, contrary as this opinion is to my former observations on the dietary’.^43^ Small amounts of meat and milk were added to the diet, and eight ounces of bread substituted for oatmeal at breakfast.

The present dietary consists of twenty ounces of bread, four ounces of cooked meat without bone, half a pint of soup, half a pint of skimmed milk, a pound of potatoes or an equivalent quantity of other vegetables, and a pint of gruel daily. I find that with this dietary the flesh of the prisoners is much firmer than formerly. The general appearances of health were very satisfactory with the old dietary, but it was found, when the prisoners left the prison and came to do hard work in the open air, that many of them broke down; but since the change in the dietary I have not had any representations of failure of the kind.^44^

It is likely that some of the prisoners referred to by Jebb had also been subject to the first of Milner’s experiments with prison dietary, which straddled the period 1848 to 1857. During this time Milner analysed the weight of 4,000 prisoners in over 44,000 individual monthly weighings, investigating weight change according to the length of time in prison, seasons, employment, and age and height.^45^ At a point where expert discussion was considering the quality as well as quantity of food as critical in weight gain or loss, Milner referred to the impact of another factor, in response to his observations that most men gained weight in the first couple of months after admission and then started to lose weight. He attributed this to the mental change that occurred after committal when prisoners recovered from the anxiety that marked the period before trial. Once imprisoned, their fate was decided, resulting in a feeling of relief, and ‘a reaction of the mind against the depression under which it had previously been suffering’.^46^ Later on the men would start to lose weight as the strain of continued punishment ‘begins to tell’, and at that point it became necessary to give extra diet. Addressing the issue of less eligibility, Milner also pointed out that it was a fallacy to suggest that prisoners were pampered and fed up in prison, and that it was necessary ‘to allow a large discretionary power, of giving extra food to a medical officer’.^47^

Milner’s experiments were part of a much wider enthusiasm for nutritional experimentation on institutional populations that had taken hold early in the nineteenth century, along with an expanding interest in the science of food, digestion, and Liebigian chemistry.^48^ With regard to prisons, as Durbach has argued, many of these studies had a limited impact on feeding prisoners, with local prison officials preferring to rely on their own experience and resources, with cost saving and disciplinary measures taking precedence over science.^49^ Milner’s pragmatic approach based on careful observation of the Wakefield prisoners, and those of other examples we cite below, confirm this. His close collaboration with Edward Smith, the renowned physiologist and expert on institutional diets, saw a division of labour whereby Smith focused on scientific analysis while Milner took charge of observations in the prison, with the primary objective of assessing the relationship between diet and ability to labour.^50^ Milner’s earlier research on the Wakefield prisoners was incorporated into a joint paper with Dr Edward Smith in 1862, and Smith alluded in a later publication in 1864 to his joint research with Milner, which measured changes in weight as well as the ‘quantity of nutritive and effete matters entering and leaving the body’, proving

that seclusion with inactivity does lessen the vital activity of the body, and causes a larger portion of the food to leave the body unused than occurs under ordinary circumstances, and hence that the ordinary diet out of prison would not suffice for the same person in prison without labour.^51^

With regard to Wakefield’s convicts, it was pointed out that they were fed a liberal and unform diet (similar to the one cited above, but with additional milk and oatmeal), employed in some form of manufacturing, and had nine hours a week of running or walking exercises. The treadwheel and crank were no longer used.^52^ Wakefield’s work with dietary adjustments was regarded as a success, and together with ensuring that the convicts spent more time in association at outdoor exercise, was credited with a decline in incidences of insanity. Already by 1852 Wakefield was praised over Pentonville for its successful governance, while Pentonville continued to be associated with high rates of mental breakdown.^53^

## The regulation of diet in Irish prisons

Experimentation with diet in other prison settings, particularly Irish prisons, occurred in a more quotidian manner than at Wakefield and Pentonville, prompted by the need to adapt to food supply, to make savings or based on assumptions about local dietary customs. Indeed many mid-century dietary adaptations in Irish convict and local prisons, rather than being experimental, were largely a response to the food crisis that followed the failure of successive potato crops during the Great Famine of 1845–1852.^54^ From the 1820s onwards, the Inspectors General of Prisons had sought the introduction of the separate system to Irish prisons, while central regulation of prison diets was formalised under the 1810 and 1826 Prisons (Ireland) Acts. The legislation listed a choice of three diets—‘bread’, ‘potato’ and ‘mixed’—from which the local authorities in charge of prisons were to select. The ‘bread diet’ consisted of two pounds of bread and a quart of new milk; the ‘potato diet’, nine pounds of potatoes, one pint of new milk, and one pint of butter milk; and the ‘mixed’ diet, eight ounces of meal for stirabout, four pounds of potatoes, one pint of new milk, and one pint of buttermilk. There was also a list of hospital ‘extras’, beef for broth and mutton.^55^

While the rationale behind the selection of these diets was not outlined, when potatoes, an essential component of general and prison diets prior to the Famine, became difficult to secure after 1846, local prison officials introduced alternative foodstuffs, notably oatmeal, not traditionally a feature of the Irish diet. Potatoes were removed from Armagh Gaol diets in 1846. The diet subsequently consisted of one pound of white bread and one pint of new milk for breakfast, and eight ounces of oatmeal stirabout and one pint of new milk for dinner.^56^ In January 1847 the prison inspectors raised concerns about the ‘low’ prison diet at the new Belfast Prison (1845), which implemented the system of separate confinement and replicated the diet of Armagh Prison. A supper consisting of two ounces of meat in gruel was added a year later. It was Belfast’s prison chaplain rather than the prison surgeon who inspected the provisions daily, and, while the prison inspector was subsequently satisfied that the diet was ‘good and sufficient’, he suspected ‘the Physician may find it too lowering’.^57^ The exchanges on the diet at Belfast not only underscore the seniority of prison chaplains, who were second only to prison governors in the early years of the separate system, and the relatively low status of the prison doctor. They also highlight tensions between prison chaplains and surgeons at individual prisons, and between central and local actors as adequate dietary provision under the separate system was decided on.^58^

Despite prevailing famine conditions, prison diets were curtailed in the 1840s, sometimes as cost cutting exercises or due to the pressure placed on prison officers to impose the principles of less eligibility. At Grangegorman Convict Depot, surgeon Dr Harty concluded that the female convicts awaiting transportation were ‘overfed’, and their diet was reduced in July 1846. They were given ‘animal food only twice in each week until 5 or 6 weeks prior to embarkation when if considered necessary by the medical officer the present dietary can be resumed’.^59^ Determined to maintain the principle of less eligibility, in 1849 prison inspectors Galway and Long authorised a sparser diet for local prisons in Ireland, convinced not only that they were more generous than workhouses and the diet of the average agricultural labourer, but also that the poor had committed crimes to secure access to food during the Famine.^60^ In England, even without famine conditions, the poor ‘were stunted, some wasted and most hungry’, undernourishment was a serious issue, and there was similar pressure to adhere to the dogma of less eligibility.^61^ Few English local prisons initially adopted Graham’s dietary, only sixty-one of 195 in 1845, and alongside adaptions to diet in individual prisons, there were regional differences, with prison diets in the north of England tending to have a higher energy value than the south. Johnston has suggested that this was due to the consumption of larger amounts of oatmeal and potatoes in northern England, while wages tended also to be higher in the north, and magistrates were less concerned that higher rations might encourage crime.^62^

The opening of the long awaited Mountjoy Convict Prison in 1850 coincided with the final years of the Great Famine and the winding down of transportation, both factors contributing to increased prisoner numbers. Unlike Pentonville, with its emphasis on selecting prisoners ostensibly in good health, Mountjoy’s convicts were reported to be in ‘debased physical condition’ from the combined effects of deprivation and spending long periods in overcrowded prisons and convict depots.^63^ This context shaped the modifications introduced to the separate system at Mountjoy, including an ‘alteration’ to the prison diet.^64^ Dr Francis Rynd, the first Medical Officer at Mountjoy, who had been Medical Superintendent at Smithfield Convict Depot during the fever outbreaks of 1848 and the 1849 cholera epidemic, also rejected large numbers of convicts as unfit for the regime.^65^

There were no significant experiments with diet during Rynd’s time at Mountjoy and, according to his successor Dr Robert McDonnell, Rynd opposed curtailing prison diets.^66^ In relation to overseeing the health of prisoners, Rynd claimed his authority at Mountjoy was ‘unrestricted’, which differentiated the regime from that at Pentonville where the chaplains were influential in matters relating to prisoners’ health. Rynd and Mountjoy’s governor had commented on the poor health of convicts and monitored convicts’ weight on a monthly basis throughout 1851, noting the number of men who had gained or lost weight and those whose weight did not change.^67^ In contrast to Milner’s findings at Wakefield, Rynd found that over 46 per cent lost weight during the first month (16 per cent ‘remained stationary’), 74 per cent then gained weight in the second month, 47 per cent in the third month, and 67 per cent in the fourth month.^68^ In his concluding observations, Rynd noted that the ‘results of the separate system of confinement have not been exactly the same at Mountjoy as elsewhere, particularly with reference to the occurrence of insanity in the prison’ and after one year of assessing convicts’ weight, he was satisfied that the Mountjoy diet was ‘good and sufficient’.^69^ At Spike Island Public Works Prison, where convicts worked in association in arduous conditions, they were provided with ‘a higher scale of dietary where necessary, which will give the convict sufficient strength of constitution to enable him to resist disease’.^70^ In 1854 Sir Walter Crofton, chair of the newly established Board of Directors of Convict Prisons for Ireland, noted the importance of diet for preventing listlessness among convicts there:

We therefore felt it to be our duty to provide that when his time of penal servitude shall have expired, he will be restored to society with an unimpaired constitution, and with sufficient health and energies to enable him to take a respectable place in the community, and engage in such industrial, [*sic*] pursuits as his moral and religious training while under our charge, will, we trust, prompt him to follow.^71^

In a similar vein, observers criticised the prison inspectors’ 1849 decision to reduce the dietary provision in Irish local prisons as a false economy, insisting that underfed prisoners with enfeebled constitutions were likely to become charges on the state as inmates of workhouses, hospitals, and prisons.^72^ As Kenneth Carpenter has argued, similar concerns were expressed in England, largely by a public concerned about the ability of prisoners to work on release rather than prison administrators who remained determined to impose regimes of deterrence on prisoners.^73^

Experiments involving dietary restrictions, however, commenced in Ireland towards the end of the 1850s. Under Crofton’s marks system, introduced in 1854, labour was treated as a privilege rather than a punishment, and convicts did not perform hard labour when on probation. A meat diet was regarded as excessive for convicts on probation, and despite Rynd’s satisfaction with the diet, in April 1858, Crofton consulted Dr Robert McDonnell, Mountjoy’s Medical Officer, to inquire whether ‘meat could either be reduced in quantity or altogether excluded’ from the diet, citing the examples of dietaries used at Belfast, which operated the separate system in full, and diet at Irish military prisons, where ‘no evil effects have resulted from the absence of meat’.^74^ By October McDonnell and Crofton had developed a meat-free ‘reception diet’ for Mountjoy, and convicts were to be placed on it during the probationary stage, the first two months of their sentence, when they were not performing hard labour.^75^ Having closely observed its effects for nearly two years, McDonnell recommended that the time period be extended to four months for ‘robust’ male prisoners, although, as discussed below, he subsequently introduced meat for convicts in weak condition.^76^

When a group of Wakefield magistrates visited Mountjoy in 1862 to assess how prisons and notably the marks system operated there, they commented positively on the decision to place convicts on the lowest diet consistent with the maintenance of health, with no meat whatsoever during the first probationary stage.^77^ In the same year Reverend W.L. Clay (son of Reverend John Clay, an early enthusiast for the separate system and Chaplain at Preston Gaol), in his review of the operation of the separate system in England and Ireland, reserved special praise for convict prisons in Ireland where the prison system had been adapted to mitigate the impact of the separate system in lowering the bodily organs and weakening the faculties.^78^ Clay described how the animal tendencies of the prisoner must be lulled to sleep by the ‘depressing power of isolation’, though careful consideration should be given to diet so as not to injure mental or physical health.

Plenty of fresh air, therefore, brisk exercise, and suitable diet, are necessary. If the diet is too low, it will turn depression into despondency; if too high, it will produce excitement and irritability. The god of criminals is their belly; and to baulk the belly-god to the utmost extent is both wise and good.^79^

Clay grumbled about the soft regime at Pentonville compared with Mountjoy, which was as ‘penal as possible’ with meagre rations, whereas the Pentonville prisoner was first taught a trade and put on ‘bounteous diet’, while prisoners on longer sentences were offered ‘belly bribes’.^80^ In Mountjoy, the mark system gave prisoners the opportunity to substantially reduce their sentences through good behaviour, and ‘the wits and will of the Irish convict are kept on the alert, and so he thrives and fattens on the spare simple diet which would not keep the model prisoner from pining and sickening under the sluggish regime of Pentonville’.^81^ Under the mark system, convicts were granted a relaxation of prison discipline at each of the three progressive stages, and in the third stage male and female convicts were allowed a ‘better diet’ as well as ‘work’.^82^

Jebb, however, was highly critical of such enthusiasm for the Irish mark system.^83^ Commenting on dietary provision, he drew out what he saw as a contrasting need among Irish and English prisoners, remarking that few of the ‘lower classes in Ireland are accustomed to meat, and will therefore thrive without it’, whereas ‘English prisoners would sink under the privation’.^84^ Jebb’s comments echoed wider beliefs that endured for several decades after the Famine, that Irish labourers not only preferred, but were physiologically more suited to, traditional food stuff and consumption patterns, and did not require the same diet as English workers to perform heavy labour.^85^ Jebb also stressed the importance of a ‘full and sufficient diet’ for prisoners ‘from the very first day of imprisonment’, especially as a tool to protect prisoners ‘against the depressing influence peculiar to imprisonment when combined with a depressed state of physical condition resulting from the want of a full diet’.^86^

While the Wakefield magistrates, Jebb and others, drew attention to Mountjoy’s ‘meat-free’ diet, according to McDonnell, who provided extensive detail on the diet in his 1865 Annual Report to the Directors, there were, in reality, two approved dietary scales for convicts undergoing cellular isolation at Mountjoy. McDonnell, unlike Jebb, rejected claims that additional diet was necessary to counteract the depressing influence of prisons, arguing that most criminals do not experience imprisonment as a degradation and consequently do not become depressed. The few that become despondent, he insisted ‘never ask for more food, and could not digest it if they got it’ as they were ‘irritable, nervous, sleepless, and out of health’.^87^ In 1858, at the request of the prison Directors, McDonnell experimented on healthy prisoners, and, he claimed, on himself, to identify reductions in the scale of the diet at Mountjoy; he subsequently developed two scales of diet for adult prisoners undergoing cellular confinement. Under diet ‘A’ prisoners received meat in their soup two days a week but not under diet ‘B’. When an audit of the cost of prisoners’ diet in 1865 revealed to the Directors, apparently for the first time, that McDonnell provided some prisoners with meat, he asserted his authority to make use of ‘one or other’ of these two scales, depending on prisoners’ medical needs, and explained how in 1859, two-thirds of prisoners were placed on the meat-free diet ‘B’ on admission while the remainder were given diet ‘A’. A rise in the incidence of scurvy in 1864, caused, McDonnell believed, by an increase in the number of prisoners undergoing punishments involving curtailed diets, prompted further dietary modifications, and McDonnell recommended that all prisoners be given meat on arrival, while prisoners on the punishment diet be provided with lime juice. While the Directors rebuked McDonnell, claiming he acted without their approval, he affirmed his authority in matters of diet as well as medicine, insisting that ‘in acting thus I have simply done my duty, and require no more sanction for the Dietary than I should for the ordering of a dose of Epsom Salts in preference to a dose of Jalap’.^88^ The dispute between the Directors and McDonnell, initially prompted by concerns over cost, thus fed into a broader set of tensions over whether the authority to determine appropriate scales of diet, and adjudicate on the health needs of prisoners, resided with the prison medical officer or with the Directors, tensions that would culminate in McDonnell’s resignation in 1867.^89^

## ‘Starving crime into surrender’: The penal era, diet and uniformity?^90^

After the 1860s English and Irish prisons moved towards a policy of nationalising prison administration, which would culminate in the 1877 Prisons Acts, and a more penal approach, although the rigour with which this was implemented varied between the two countries. The focus of separate confinement shifted from reform to punishment, with greater emphasis centrally on the uniform enforcement of hard labour and strict adherence to dietary scales.^91^ Notwithstanding the goal of uniformity, consideration of the balance between proper nutrition, and ability to comply with the prison regime, particularly in terms of labour, and the principle of less eligibility and cost formed the basis of the many debates on dietary and practices in prisons during the second half of the nineteenth century.

Dietaries, devised in the 1860s and the 1870s, tended to be assessed as being ‘*more* or *less* generous’ than Graham’s scale.^92^ Graham had warned that the diets were not to be made an instrument of punishment, and in evidence to the 1864 inquiry into the diets of convict prisons, Milner, reflecting on the experiences of Millbank in 1822, Wakefield in 1849, and a more recent reduction in 1862–63 in the dietary of prisoners at Wakefield, cautioned against reductions in the dietary that were being trialled at Pentonville.^93^ However, as Priestley has shown, many prisoners believed the diets were adjusted to test the limits of their well-being.^94^ Their memoirs testify to how acutely aware they were of the impact of prison diet on health, and many inmates noted how they rapidly lost weight and tone in prison, vividly recording the anxiety that left them unable to eat and the violent digestive disorders and illnesses that they related to poor nutrition. Jabez Spencer Balfour lost two stone on his admission to Portland Prison, which he attributed to the diet and ‘the mental torture I was enduring’.^95^ Another prisoner described how his ‘health began to utterly break down’.^96^ Poor diet also contributed to the general misery of prison life: ‘No other event in the prison day…was so keenly anticipated nor so soon after so often regretted as the arrival of food at the door of the cell.’^97^

Amidst demands for greater uniformity in prison discipline and for a more punitive prison regime, witnesses to the 1863 Carnarvon Committee sought reductions to prison dietaries. Concerns about prisoners becoming too enfeebled and physically incapacitated to perform labour and to work on release, and the threat of epidemic disease outbreaks in prisons, however, prompted the Committee to defer proposals for a national, uniform dietary scale for local prisons.^98^ Instead, they recommended ‘experiments’ on prison diets to establish how the dietary could be reduced safely, a task led by Dr William Guy of Millbank Prison and supported by Dr Maitland at Gosport Military Prison and Dr Clarke of Dartmoor.^99^ Anne Hardy has described how Guy, with his background in public health, was particularly determined to adhere ‘to a stern disciplinary diet despite criticism on medical grounds from both within and without the prison service’, pointing out that his view diverged from many of his medical colleagues working in prisons.^100^ While Guy proposed removing meat from dietaries to the Carnarvon Committee and suggested that convict dietaries were excessive in contrast to local prisons, Martin Wiener has argued that he was eager to develop prison health policies based on scientific knowledge and defended the authority of medical officers to alter diet on grounds of health.^101^ Guy thus ‘bridged the medical and penal worlds of nineteenth-century Britain’, and represented the conflicts that other prison medical officers might face in their day-to-day work.^102^

The 1863 Commission on Transportation and Penal Servitude was less circumspect than the Carnarvon Committee, recommending that the policy of not providing prisoners meat during the first months in separation, as practised in the Irish convict system, be extended to English convict prisons. While they did not advocate for the reduction of diet for convicts working in association in public works prisons, they also suggested some experimentation ‘to ascertain whether any reduction can safely be made’.^103^ In his evidence, Walter Crofton advocated that a meat free diet could be maintained up to one month prior to a convict’s removal to an intermediate public works prison.^104^ In contrast, there were concerns that the diet in Irish local prisons was too sparse, and in 1868 a medical committee appointed to inquire into dietary scales in Irish county and borough gaols found that 82 per cent of prison governors and 94 per cent of surgeons regarded the prison diet as insufficient.^105^ Hard labour, however, was not carried out in Irish gaols with much rigour, and prisoners performing work were regularly provided with extra rations to counteract any deleterious effects.^106^

A key aim of the 1877 Prisons Acts was to calibrate adequate provision of food for prisoners working at hard labour, under the more punitive and disciplinary regimes introduced by the legislation to convict and local prisons. In 1878 the Committee on Prison Dietaries, known as Du Cane’s Scientific Committee, was charged with considering whether the amended prison discipline necessitated new dietary scales, with ‘health, disciplinary effects and deterrent properties’ given equal weight.^107^ Du Cane’s committee concluded that prisoners not working at hard labour, including women, should be placed on a sparser diet. Accepting the principle that diet should be varied according to length and type of sentence, a staged dietary system was advocated.^108^ Du Cane’s recommendations, swiftly confirmed as prison rules for England, detailed a range of dietary scales for various classes of prisoners in local prisons. These were sparser than the 1843 and 1864 diets, especially for prisoners not performing hard labour or on short sentences.

These numerous investigations and inquiries into prison diets in England and Ireland revealed the extensive use by prison doctors of discretionary powers to approve dietary extras for medical reasons, powers authorised by the prison regulations. In 1863 Guy had claimed that dietary extras, introduced ‘for temporary reasons’, frequently became permanent, resulting in significant variation in diet across the prison estates.^109^ Du Cane’s committee also showed that the 1843 and 1864 dietary scales were regularly altered by prison officials and that prison doctors frequently authorised increased quantities of food. In 1878 only twenty-six of 114 local prisons in England followed the dietary of the 1864 committee.^110^ The 1867 investigation into practices in Ireland also pointed to variations, reporting that 215 of the 230 prisoners in Richmond Bridwell received extra food.^111^ While critical of the overuse of discretionary powers, Du Cane’s committee reaffirmed the importance of the prison medical officer in adjudicating prisoners’ dietary needs and recommended that they retain discretionary powers to approve extras.^112^ However, while noting that diet should not be diminished where health was damaged, they cautioned against prison doctors allocating too liberal a diet.^113^ The General Prisons Board’s 1880 Medical Commission in Ireland, established to inquire into the suitability of the 1878 English prison diet for Irish prisons, came to a similar conclusion, though they disapproved ‘of any interference with the dietary scales as laid down for healthy prisoners’.^114^ Dietary privileges in the convict system, including those allowed at Mountjoy Convict Prison, had been abolished by 1878 and the reduced diet for convicts during the first three months of their sentences enforced.^115^ Despite these efforts to enforce uniformity, the 1880 Irish Medical Commission and the 1884 Royal Commission on Irish Prisons again revealed significant variations in dietary practices and high levels of intervention. Medical officers were accused of over prescribing improved diets as a prophylactic against illness among ‘juvenile offenders, nursing mothers, and aged prisoners’ who, the Commissioners claimed, were ‘in excellent health’.^116^

## Diet as a tool of health and punishment

As well as authorising additions to ordinary diets, prison doctors were permitted to sanction hospital diets. Prisoners suspected to be in poor physical and mental health or considered unable to withstand the prison regime were moved to the prison hospital where a more liberal diet was provided. After 1877, the ordinary hospital diet in local prisons in England and Ireland for men and women was set at sixteen ounces bread, five ounces of cooked mutton, eight ounces of potatoes, and eight ounces of rice pudding, with thirty ounces of tea. There was also an ‘extra’ hospital diet, which included additional bread and mutton, and a ‘low’ hospital diet of eight ounces of bread only. Hospital diets also included tea, twenty ounces of arrowroot made with milk, and another twenty ounces of milk and other ‘extras’ including fortifying spirits such as wine and porter .^117^ When concerns were raised about prisoner Balfour’s rapid decline in health and weight after admission to Portland Prison, he was moved to the prison hospital and declared the food there ‘excellent’.^118^ He was later transferred to Pentonville where he spent four months in the hospital, which, he affirmed, had its own kitchen and a good diet.^119^

While the decades after nationalisation were characterised by punitive regimes, including reduced dietaries, and efforts to enforce greater uniformity across the prison estates, medical officers devoted significant time to assessing prisoners’ physical condition and capacity to perform hard labour and, in some instances, suggested dietary interventions. At Dartmoor, Dr R.E. Power, who was responsible for nearly 1,000 convicts, argued for a more generous diet than prisoners held elsewhere were given.^120^ Given the severe conditions, especially for prisoners working on the moorland in harsh weather, Power noted how Dartmoor prisoners lost large amounts of weight, were frequently admitted to hospital, and provided ‘enormous’ amounts of cod liver oil, estimated between sixty and eighty gallons a year, as a food supplement.^121^ Prisoners themselves recounted the toll that performing hard labour while on a prison diet took on both body and mind. ‘One Who Has Tried It’, describing his experiences of an English local prison in the 1890s, recalled intense feelings of hunger after his first day on the treadwheel:

This, my first spell on the wheel, lasted for a couple of hours. I had done the work on a very small breakfast, and I felt quite crushed when I returned to my cell…Dinner was served almost immediately…and although I absolutely ‘wolfed’ the bread and potatoes, I could have eaten three times the amount, and was still starving when done.^122^

He was subsequently placed on punishment diet for refusing ‘the wheel’. His ‘brooding fever of disgust and annoyance’, however, prompted the prison medical officer to admit him to hospital, where he remained ‘in bad ways for some days’.^123^

During the 1880 General Prisons Board survey of diets in Irish local prisons, expert opinion submitted by medical officers as to the suitability of the dietaries introduced in 1878, especially for prisoners performing labour, highlighted the damage inflicted on prisoners’ mental as well as physical health. They also complained that the dietaries were overly complicated and time-consuming to implement, adding to doctors’ workloads. Dr Kelly at Drogheda local prison noted that ‘when continued for a longer period than one month’, class 1 and 2 diets ‘produce…“melancholia” in the average male prisoner’, but not among female prisoners, as the ‘labour of the female prisoners is not being of so arduous a nature as that of the male’.^124^ Dr H. Minchin at Grangegorman described the class 1 diet as ‘quite sufficient’ for seven days. Worryingly, those committed for longer terms ‘begin very soon to exhibit the depressing effects of a restricted diet, and suffer from languor, debility, indigestion, etc.’, terms similar to those applied in Pentonville in the 1840s. In such cases Minchin prescribed milk and an increase in bread and potatoes.^125^ The Galway prison doctor, Dr R. Kinkead considered the ‘standard of the diet too low on general principles’ and ill-suited for a ‘badly fed and poorly nourished’ population:

Many are broken down by drink, exposure, and debauchery, and cannot well stand a low scale of diet especially when the depression of imprisonment, the solitude of their cells, and the hard labour of the treadwheel is taken into account.^126^

Some doctors, seemingly rejecting the principle of less eligibility, insisted that prisoners needed more rather than less food when first committed.^127^ When the new diet was first introduced in 1878, Dr J. Moore at Belfast Prison, in comments that echoed older theories of constitutional medicine, observed a marked ‘lowering of vitality’ among prisoners, referring to: ‘the look of dullness and depression, the absence of elasticity in the step when at exercise or going to labour’, which gave the ‘inmates the appearance of a famine-stricken population’. Meanwhile, the number of prisoners discharged on medical grounds increased.^128^ The 1880 survey also brought attention to the regularity with which doctors prescribed extra diet rations; at Galway ninety-three prisoners were in receipt of extra food allowances in addition to those prisoners in the hospital on enhanced rations, while at Wexford prison the diet was supplemented to ‘prevent debility, whitlows and boils supervening’.^129^

Diet used as a tool of discipline might tip over into extreme cruelty, resulting in prisoners’ illness or even death. The regulations on the duration and frequency of punishment (i.e. bread and water) diets were often flouted, and many cases of abuse through food withdrawal were reported. An 1846 anonymous memorial to the Irish prison inspectors about Omagh Gaol Hospital alleged that the prison surgeon confined prisoners to the hospital for ‘twenty to thirty weeks, stripped them of clothing, and deprived them of medicine’. The memorialists claimed that prisoners were ‘half starved with hunger’ and that the medical attendant, Dr Maxwell, practised ‘great and glaring cruelties’, citing specific examples of prisoners who had died from hunger and thirst.^130^ Two notorious instances of cruelty occurred in Birmingham and Leicester Gaols in the 1850s, involving dangerous restriction of diet. Excessive infliction of crank labour and failure to complete the allocated tasks resulted in floggings and the withdrawal of food, culminating in several suicides. One Birmingham prisoner, ‘the boy Andrews’, aged fifteen, was repeatedly placed on a bread and water diet for failing to complete his crank work and for ‘injuring’ the crank. He was put in a leather collar and straitjacket and had water thrown over him before he hanged himself in his cell.^131^ The surgeon at Birmingham, Mr Blount, described Andrews as ‘lazy and sullen’: ‘He never said that he was not strong enough to work the crank.’ Blount was considered to have acted illegally in failing to protect those under his charge, several of whom were very young and of ‘unsound mind’.^132^

High profile allegations of cruelty relating to prison diet emerged at Armagh Gaol shortly after nationalisation. Two prisoners, Patrick Grimes, aged forty-six, and Margaret Girvan, aged forty-nine, died in June and July 1879. During the inquest into Girvan’s death, the jury found the ‘very low scale of diet’ at Armagh gaol to be a contributing factor.^133^ Girvan, described as a vagrant whose constitution was ‘shattered’ by the time of her death, had been confined on fifty occasions.^134^ At the subsequent inquest, it was noted that the prison dietary had not been changed after the death in June of Patrick Grimes. Grimes, serving a sentence of six months hard labour, entered the prison in ‘delicate health’ and was certified as unfit for hard labour. For the first month, he received the normal meat-free prison diet, but by May 1879 he was in the prison hospital with a fever. The prison surgeon Dr Palmer, who considered the hospital diet insufficient, provided Grimes with beef, eggs, and milk and informed the General Prisons Board and the Lord Lieutenant’s office that he considered Grimes’ life to be ‘in immediate danger by further confinement’. Grimes subsequently died of heart disease and ‘other ailments’, notably inflammation of the lungs. At the inquest, while the jury concluded that the treatment the prisoner received in Armagh prison was ‘humane and proper’, they condemned the ‘new scale of diet and attendance to sick in hospital, which has deprived prisoners of the new-milk diet, and substituted a food not sufficient for the support of life and proper attendance’.^135^ Grimes’ case was covered in the press in Britain and Ireland, notably when it emerged in the House of Commons that six of the jurors had been convicted prisoners.^136^ Medical commentary on the case explicitly linked prison deaths caused by pneumonia, including that of Grimes’, to the low diet, while Dr T. Wemyss Bogg, ex-medical officer at Louth Prison, cautioned that the effect of prolonged, sparse diet ‘must be still further to depress the mental and physical energies’. ‘Low diet is an edge-tool’, he observed, ‘unfit for indiscriminate use, only to be employed in cases of absolute necessity by skilful hands and under careful supervision’.^137^

There was considerable debate on the safe application of the prison punishment diet of bread and water. For McDonnell at Mountjoy, the ‘punishment diet’ posed a significant danger to both the physical and mental health of convicts. He continued to ‘interfere’ with the diets of prisoners undergoing punishments throughout 1865 and 1866 and argued that punishments, including punishment diet, experienced as unfair or unjustified could prompt mental irritation and attempted suicide among convicts.^138^ Much of the debate focused on how long or how often prisoners could withstand the punishment diet, while at Birmingham Gaol the governor seemed unaware of what the rules were.^139^ In the 1850s convicts in Ireland were normally on punishment diets for three days, but in exceptional cases they were placed on such diets for much longer periods.^140^ By the late nineteenth century, the usual period of punishment was three days, although the Kimberley Commission found that there was no limit on the number of successive punishments, so long as these were broken by one day on the ordinary diet.^141^ Du Cane’s 1878 committee regarded the limit of three days on dietary punishments as too restrictive, as well as disapproving of the hospital diets as too liberal and too frequently allowed. Seeking a dietary punishment that could be ‘measured by weeks rather than by days’, the committee devised a ‘stirabout’ punishment diet, and a ‘full’ version was provided for hard labour prisoners. In introducing these scales, it sought ‘not a starvation diet, but a diet which will supply the requisite amount of nutriment while ministering in the least possible degree to such gratification as is to be derived from the ingestion of food’.^142^ Defending the disciplinary regime, the Committee argued that diseases related to defective nutrition were brought into prisons while those ‘of mal-nutrition are even arrested by imprisonment’.^143^ They also concluded that in a large number of cases, imprisonment, as now generally conducted, is a condition more or less akin to that of ‘physiological rest. The struggle for survival is suspended…Tranquillity of mind and freedom from anxiety are leading characteristics of his life. From the moment that the prison gates close behind him, the tendency, in most cases, is to lessened waste of tissue; he lives, in fact, less rapidly than before.’^144^

## Conclusion

Prison diet and its management was recognised as being just as crucial in the early years of separate confinement, with its emphasis on reform, as in the penal era that set in after the 1860s, involving hard labour, harsh conditions, and restricted diet, and prison officials, doctors, and administrators remained preoccupied with the relationship between diet and separate confinement, even after the probationary period was reduced to periods of less than seven months in the 1860s. Debates and decisions determining prison dietaries were shaped by concerns of cost, the principle of less eligibility and by emerging scientific discourses, though the impact of the latter on practical implementation of dietary provisions remained unclear. Our research reveals additional factors that prison medical officers brought to bear when formulating prison diet. Under the separate system, prison administrators and prison doctors in both countries asserted the importance of food to psychological wellbeing, and in turn the ability of the prisoner to withstand cellular isolation and to reform, though this had additional resonance in Ireland under famine conditions. In England, this emphasis appears to have been lost to a certain extent when imprisonment became more about punishment and uniformity than reform after the 1860s. Indeed, some prison officials and medical officers emphasised the psychological rest that ensued on entering prison and prisoners’ consequent ability to manage with less food, though others, like Milner, advocated caution given the special conditions of separate confinement. As late as the 1880s, some prison doctors in Ireland persisted in highlighting the relationship between diet and mental distress, which resonated with earlier ideas about constitutional medicine.

Exploring the management of diet in mid-nineteenth-century prisons offers a powerful lens for exploring the challenges facing prison medical officers more broadly during this period, including their responses to the pressures put on them to support prison discipline and the penal system, on the one hand, and, on the other hand, to maintain the health of the inmates under their charge. Despite official proclamations that diet should not be used to impose punishment and that the prisoner should not be subjected to treatment that worsened his or her health, many prisoners suffered massive dietary deprivation resulting in physical and mental harm, exacerbating their experiences of incarceration. However, our evidence contests Sim’s conclusion that prison medical officers were unable to act independently or benevolently, as in many instances they adjusted diet to benefit the wellbeing of the prisoners and thus undermined core objectives of penal administration such as uniformity, and, like Milner and McDonnell, asserted their authority to do so.

The scale on which prison doctors exerted their authority and disrupted the implementation of punitive rations—in terms of the number of prisons affected and their impact in individual prisons—was at times remarkable. The numerous official investigations, intended to enforce uniformity of practice in Irish and English prisons, repeatedly revealed instances of prison doctors’ adaptions and interventions. Many prison doctors adjusted prisoners’ diets to ameliorate their conditions and safeguard their health. Others imposed further limitations, either in the interests of experimentation or apparently, in the cases of cruelty and neglect cited above, because it was in their power to do so. In both scenarios, this offered opportunities, as Milner and MacDonnell clearly asserted, to express authority and expertise in dietary management. The result was dramatic variation in regulating diet, both within and across the English and Irish prison estates, according to interpretations of the rules and manipulation of official prison dietaries and the energy and assertiveness of the prison medical officer. Many of these adjustments took the form of experiments, which may have been more common in English than in Irish prisons, on large numbers of prisoners without their consent. These adjustments might have been shaped by science and new knowledge of nutrition and physiology, but, more typically, had taken the form of quotidian experiments to improve the welfare of prisoners, to save prison resources, or were based on assumptions about local dietary customs. While the spurs driving dietary interventions could and did differ in Ireland and England, given the distinctive conditions of the labouring poor in those countries, our evidence suggests that many of the responses of Irish and English medical officers were not only pragmatic but similar in nature.

Confirming Durbach’s arguments, our evidence on individual prisons demonstrates that local considerations often trumped national regulations, resulting in tensions between national inspectors and local prison doctors. In addition, pragmatic considerations related to health and the ability to conform to prison regimes might also have trumped scientific findings. While the quality of food was debated by experts in many prisons, it was the quantities and types of foodstuffs that were more typically adjusted in response to concerns about the declining health and weight of prisoners. Our sources indicate that weight loss was common, although explained in a variety of ways by prison doctors, and some dietary adjustments left prisoners vulnerable to disease or illness. Even in Wakefield, with its relatively liberal dietary, Milner observed that the weight of prisoners was below that of persons of similar age and height in a state of freedom, something regarded as ‘the normal condition of prison discipline’.^145^ This is contrary to the assertions of Price and Godfrey, that weight loss was relatively uncommon amongst prisoners. Prison diet also left them hungry. Michael Davitt, though moved during his second stretch of imprisonment to Portland Prison infirmary where he was given a special diet, recorded his reflections on food and prison diet in 1898: ‘There is no bodily punishment more cruel than hunger…that remorseless, gnawing, human feeling which tortures the mind in thinking of the sufferings of the body.’^146^

## Footnotes

1

Helen Johnston , Crime in England 1815–1880 (New York: Routledge, 2015), 96, 94 .

2

James Vernon , Hunger: A Modern History (Cambridge, Mass. and London: Harvard University Press, 2007), 160 .

3 See e.g. Johnston, *Crime in England*, *op. cit.* (note 1); Helen Johnston , Barry Godfrey , and David J. Cox , Penal Servitude: Convicts and Long-Term Imprisonment, 1853–1948 (Montreal: McGill-Queen’s Press, 2022); Seán McConville , English Local Prisons 1860–1900: Next Only to Death (New York: Routledge, 1995).

4 For the establishment of prison dietaries, see Kenneth J. Carpenter , ‘Nutritional Studies in Victorian Prisons’, Journal of Nutrition, 136 (2006), 1–8 16365049 10.1093/jn/136.1.1; M. Heather Tomlinson , ‘
*“*Not an Instrument of Punishment”: Prison Diet in the Mid-Nineteenth Century’, *Journal of Consumer Studies & Home Economics*, 2 (1978), 15–26 ; McConville, *English Local Prisons 1860–1900*, *op. cit.* (note 2), esp. ch. 7; Johnston, Godfrey and Cox, *Penal Servitude, op. cit.* (note 2), 109–15; Catherine Cox and Hilary Marland, *Disorder Contained: Mental Breakdown and the Modern Prison in England and Ireland, 1840–1900* (Cambridge: Cambridge University Press, 2022), 88–99; Ian Miller , ‘Food, Medicine and Institutional Life in the British Isles, c.1790–1900’, in Carol Helstosky (ed.), The Routledge History of Food (London: Taylor & Francis, 2014), 200–19.

5 Cox and Marland, *Disorder Contained*, *op. cit.* (note 4), 204.

6

Seán McConville , A History of English Prison Administration. Vol. 1. 1750–1877 (London, Boston, and Henley: Routledge & Kegan Paul, 1981), 357.

7
*Ibid.*, 256–9; William James Forsythe, *The Reform of Prisoners 1830-1900* (London: Croom Helm, 1987), ch. 8.

8 Forsythe, *Reform of Prisoners, op.cit.* (note 7), esp. ch. 3; Anne Hardy , ‘Development of the Prison Medical Service, 1774-1895’, in Richard Creese, W.F. Bynum , and J. Bearn (eds), The Health of Prisoners: Historical Essays (Amsterdam and Atlanta, GA: Rodopi, 1995), 59–82 9061250.

9

Ciara Breathnach , ‘Medical Officers, Bodies, Gender and Weight Fluctuation in Irish Convict Prisons, 1877–95’, Medical History, 58 (2014), 67–86.24331215 10.1017/mdh.2013.72PMC3866701

10

Joe Sim , Medical Power in Prisons: The Prison Medical Service in England 1774–1989 (Milton Keynes and Philadelphia, PA: Open University Press, 1990), 42–3; Miller, ‘Food, Medicine and Institutional Life’, *op. cit.* (note 4), 200–19. See also Martin Wiener, *Reconstructing the Criminal: Culture, Law and Policy in England, 1830–1914* (Cambridge: Cambridge University Press, 1990); Cox and Marland, *Disorder Contained*, *op. cit.* (note 4), ch. 3; Philip Priestley, *Victorian Prison Lives* (London: Pimlico, 1985), for the operation of dual loyalty and role of the prison medical officer in the Victorian Prison.

11

Nadia Durbach , Many Mouths: The Politics of Food in Britain from the Workhouse to the Welfare State (Cambridge: Cambridge University Press, 2020), ch. 1; Tomlinson, ‘*“*Not an instrument of punishment”’, *op. cit.* (note 4); Felix Driver, *Power and Pauperism: The Workhouse System, 1834–84* (Cambridge: Cambridge University Press, 1993), 22–6. Durbach has also investigated the ways in which the dietary of juvenile offenders was experimented with around 1900, as the importance of improving the nourishment of youth in the interests of the state overrode the principle of less eligibility: Nadia Durbach, ‘Feeding “Growing Boys” and Nourishing “Handy English Lads”: British Prison Diets and the Reclamation of the Male Juvenile Offender, 1895–1908’, *Gender & History*, 34 (2022), 632–47.

12 For prison doctors’ interventions on diet before 1850, see Peter McRorie Higgins , Punish or Treat? Medical Care in English Prisons 1770–1850 (Victoria, BC: Trafford, 2007), ch. 7. For anxieties around institutional feeding and changing ideas on food in workhouses, see Ian Miller, ‘Feeding in the Workhouse: The Institutional and Ideological Functions of Food in Britain, ca. 1834–70’, *Journal of British Studies*, 52 (2013), 940–62. See also Valerie J. Johnston, *Diet in Workhouses and Prisons, 1835–1895* (New York: Garland, 1985); E. Margaret Crawford, ‘The Workhouse Diet in Ireland Before and During the Great Famine’, in John Burnett and Derek J. Oddy (eds), *The Origins and Development of Food Policies in Europe* (London and New York: Leicester University Press, 1994), 207–22.

13 See William Murphy , Political Imprisonment and the Irish, 1912–1921 (Oxford: Oxford University Press, 2014); William Murphy, ‘Dying, Death and Hunger Strike: Cork and Brixton, 1920’, in James Kelly and Mary Ann Lyons (eds), *Death and Dying in Ireland, Britain, and Europe: Historical Perspectives* (Dublin: Irish Academic Press, 2013), 297–316; Ian Miller, *A History of Force Feeding: Hunger Strikes, Prisons and Medical Ethics, 1909–74* (Cham: Palgrave Macmillan, 2016); Ian Miller, ‘“A Prostitution of the Profession”? Forcible Feeding, Suffrage and Medical Ethics, 1909–14’, *Social History of Medicine*, 26 (2014), 225–45; Kevin Grant, *Last Weapons: Hunger Strikes and Fasts in the British Empire* (Berkeley, CA: University of California Press, 2019).

14

Kim Price and Barry Godfrey , ‘Victorian Systems will not Solve Modern Prison Health Problems’, Lancet, 393, 10169 (26 January 2019), 312–13, p.31310.1016/S0140-6736(19)30112-630696563. Ian Miller is persuasive in identifying the methodological pitfalls of applying current definitions of nutrition and nutritional needs to inmates of nineteenth-century institutions in Miller, ‘Feeding in the Workhouse’, *op. cit.* (note 12). For measuring prisoners’ weight, see Sara Horrell, David Meredith, and Deborah Oxley, ‘Measuring Misery: Body Mass, Ageing and Gender Inequality in Victorian London’, *Explorations in Economic History*, 46, 1 (2009), 93–119; Breathnach, ‘Medical Officers’, *op. cit.* (note 9).

15

David Meredith and Deborah Oxley , ‘Food and Fodder: Feeding England, 1700–1900’, Past and Present, 222, 1 (2014), 163–214 .

16 Carpenter, ‘Nutritional Studies in Victorian Prisons’, *op. cit.* (note 4), 1; McConville, *A History of English Prison Administration*, *op. cit.* (note 6), 144–5.

17 McConville, *A History of English Prison Administration*, *op. cit.* (note 6), 145–6.

18 Michael Ignatieff, *A Just Measure of Pain: The Penitentiary in the Industrial Revolution* (London and Basingstoke: Macmillan, 1978), 176. Eighteen years later there was another outbreak of scurvy at Millbank, related by the prison doctor William Baly to the absence of potatoes in the diet of the affected prisoners. Following the addition of potatoes, no further cases were reported. William Baly, ‘On the Prevention of Scurvy in Prisons, Pauper Lunatic Asylums, etc.’, *London Medical Gazette*, new series, 1 (1842–43), 699–703. See Laura Mary Sellers, ‘Managing Convicts, Understanding Criminals: Medicine and the Development of English Convict Prisons, c.1837–1886’ (University of Leeds PhD, 2017), ch. 1 for William Baly’s work at Millbank.

19 See for experimentation in prisons, Miller, *A History of Force Feeding*, *op. cit.* (note 13), 207–8; Susan Lederer , Subjected to Science: Human Experimentation in America before the Second World War (Baltimore, MD: Johns Hopkins University Press, 1995)11618486; Allen M. Hornblum, *Acres of Skin: Human Experiments at Holmesburg Prison* (New York: Routledge, 1998).

20 See Catherine Cox and Hilary Marland , ‘“He Must Die or Go Mad in This Place”: Prisoners, Insanity, and the Pentonville Model Prison Experiment, 1842–52’, Bulletin of the History of Medicine, 92, 1 (2018), 78–109 29681551 10.1353/bhm.2018.0004PMC5944833.

21 John T. Burt, *Results of the System of Separate Confinement: As Administered at the Pentonville Prison* (London: Longman, Brown, Green, and Longmans, 1852), 158.

22 Second Report of the Commissioners for the Government of the Pentonville Prison, 1844 [536], 10.

23 The National Archives (TNA), PCOM 2/84, Pentonville Prison: Minute Books, Meeting of 17 December 1842, 98.

24 Second Report of the Commissioners, Appendix E, *op. cit.* (note 22), 53.

25
*Ibid.*, 10–11.

26
*Ibid.*, 11.

27 Third Report of the Commissioners for the Government of the Pentonville Prison, 1845 [613], 19–20.

28 Cox and Marland, ‘“He Must Die or Go Mad in This Place”’, *op. cit.* (note 20), 85–7.

29 J.G. Malcolmson, ‘On the Effects of Solitary Confinement upon Health’, *Lancet*, 28, 712 (27 April 1837), 163–5, p.163.

30 Christopher Hamlin, ‘Could You Starve to Death in England in 1839? The Chadwick-Farr Controversy and the Loss of “Social” in Public Health’, *American Journal of Public Health*, 85, 6 (1995), 856–66, pp.860, 861.

31
*Extracts from a Report on Pentonville Prison, by Sir Benjamin Brodie and Dr. Ferguson*, dated January 1851. Cited in Burt, *Results of the System of Separate Confinement*, *op. cit.* (note 21), 270; Hamlin, ‘Could You Starve to Death?’, *op.cit.* (note 30), 861.

32 Fifth Report of the Commissioners for the Government of the Pentonville Prison, 1847 [818], 14.

33 TNA, Home Office (HO) 45/1451, Lunacy; Poor Law and Paupers; Prisons and Prisoners, Convict Department at Wakefield, J. Jebb to Home Office, 6 Jan. 1849, Memo by Lieut Colonel Jebb in reply to Sir George Grey’s queries on Mr Hill’s letter of 18 Dec. 1848.

34 Burt, *Results of the System of Separate Confinement*, *op. cit.* (note 21), 58.

35 Report from the Select Committee of the House of Lords on the Present State of Discipline in Gaols and Houses of Correction [Carnarvon Committee], 1863 [499], 120.

36
*Ibid.*, 124, 126.

37 Wakefield Record Office, QS 10/56 Quarter Sessions Order Book, Oct. 1846–April 1850, Wakefield Adjourned Sessions, Surgeon’s Report, 9 Dec. 1847, 98.

38 TNA, HO 45/1451, Memo by Lieut Colonel Jebb in reply to Sir George Grey’s queries on Mr Hill’s letter of 18 Dec. 1848.

39
*Ibid.*

40
*Ibid.*

41 TNA, HO 45/1451, Letter from Frederic Hill to Right Hon Sir George Grey, Bart, Whitehall, 18 Dec. 1848.

42
*Ibid.*, Convict Department at Wakefield, J. Jebb to Home Office, 6 Jan. 1849 (original underlined). See also Johnston, *Diet in Workhouses and Prisons*, *op. cit.* (note 12), 74.

43 Fifteenth Report of the Inspectors of Prisons of Great Britain. II. Northern and Eastern Districts, 1850 (1167), 77–8.

44
*Ibid.*, 78.

45 W.R. Milner, *On the Influence of the Seasons of the Year, Employment, Period of Imprisonment, etc., on the Gain or Loss of Weight, by the Prisoners Confined in the Convict Prison at Wakefield, Between January 1, 1848, and December 31, 1857* (London: no publisher, 1859), reprinted from the *Sanitary Review*, January 1859, 2.

46
*Ibid.*, 5

47
*Ibid.*, 9.

48 Miller, ‘Food, Medicine and Institutional Life’, *op. cit.* (note 4), 201. See also Ian Miller , A Modern History of the Stomach: Gastric Illness, Medicine and British Society, 1800–1950 (London: Pickering and Chatto, 2011).

49 Durbach, *Many Mouths*, *op. cit.* (note 11), 52, 53.

50
*Ibid.*, 52. Kenneth Carpenter, ‘Edward Smith (1819–74)’, *Dictionary of National Biography*: https://doi.org/10.1093/ref:odnb/25794 [accessed 12 July 2022]; Carleton B. Chapman, ‘Edward Smith (?1818–1874) Physiologist, Human Ecologist, Reformer’, *Journal of the History of Medicine and Allied Sciences*, 22, 1 (1967), 1–26; T. Cardwell Barker , Derek J. Oddy , and J. Yukdin , The Dietary Surveys of Dr Edward Smith, 1862–3: A New Assessment (London: Staples Press, 1970).

51

E. Smith and W.R. Milner , ‘Report on the Action of Prison Diet and Discipline on the Bodily Functions of Prisoners’, Part 1, Report of the Thirty-First Meeting of the British Association for the Advancement of Science, Held at Manchester in September 1861 (London, 1862), 45–81 ; Edward Smith, ‘On Private and Public Dietaries’, *Journal of the Society of the Arts*, 12, 587 (19 February 1864), 212–24, 217.

52 Smith and Milner, ‘Report on the Action of Prison Diet and Discipline’, *op. cit.* (note 51), 49. In his work on workhouse diet, Smith also emphasised the link between diet and inmates’ mental health, see Miller, ‘Feeding in the Workhouse’, *op. cit.* (note 12), 959.

53 Report on the Discipline and Management of Convict Prisons, 1852 (1852–53) [1659], 6–7, 9.

54 Cormac Ó Gráda, *Black ‘47 and Beyond: The Great Irish Famine in History, Economy and Memory* (Princeton: Princeton University Press, 2000); Peter Gray, *Famine, Land and Politics: British Government and Irish Society 1843–50* (Dublin: Irish Academic Press, 1999); Emily Mark-FitzGerald, Ciarán McCabe, and Ciarán Reilly (eds), *Dublin and the Great Irish Famine* (Dublin: UCD Press, 2022).

55 Prisons (Ireland) Act, 1810 (50 Geo. III, c.103); Prisons (Ireland) Act, 1826 (7 Geo. IV, c.74).

56 Twenty-fifth Report of the Inspectors-General on the General State of the Prisons of Ireland (RIGPI), 1846 with appendices, 1847 (805), 37.

57 Twenty-sixth RIGPI, 1847 with appendices, 1847–48 (952), 47. Ian Miller, *Reforming Food in Post-Famine Ireland: Medicine, Science and Improvement, 1845–1922* (Manchester: Manchester University Press, 2015), 74–5.

58 Twenty-sixth RIGPI, 1847, 34–5. See Cox and Marland, *Disorder Contained*, *op. cit.* (note 4), 60–3, 76.

59 National Archives of Ireland (NAI), Government Prison Office (GPO)/XB/3, Convict Prisons Minutes, 1846–1849, 3.

60 Miller, *Reforming Food*, *op. cit.* (note 57), 75.

61 Meredith and Oxley, ‘Food and Fodder: Feeding England’, *op. cit.* (note 15), 165.

62 Johnston, *Diet in Workhouses and Prisons*, *op. cit.* (note 12), 48*;* Valerie Johnston , ‘The Diets of the Local Prisons 1835 to 1878’, in Derek J. Oddy and Derek S. Miller (eds), Diet and Health in Modern Britain (London: Croom Helm, 1985), 207–30, p.217.

63 Annual Report of Inspectors of Government Prisons in Ireland for 1851, 1852–53 (1634), 37.

64
*Ibid.* See, for more detail, Tim Carey, *Mountjoy: The Story of a Prison* (Cork: Collins, 2000), 54–5.

65 Annual Report of Inspectors of Government Prisons in Ireland for 1851, 37.

66 Sixth Report of Directors of Convict Prisons in Ireland (RDCPI), 1859 with appendices, 1860 (2655), 22.

67 Annual Report of Inspectors of Government Prisons in Ireland for 1851, 53.

68 Annual Report of the Inspectors of Government Prisons in Ireland for 1850, 1852 (1429), 61.

69
*Ibid.*, 63; Annual Report of Inspectors of Government Prisons in Ireland for 1851, *op.cit.* (note 63), 53.

70 First Report of the Directors of Convict Prisons in Ireland, 1854 with appendices, 1854–55 (1958), 19.

71
*Ibid.*

72 Miller, ‘Food, Medicine and Institutional Life’, *op. cit.* (note 4), 211.

73 Carpenter, ‘Nutritional Studies in Victorian Prisons’, *op. cit.* (note 4), 1.

74 NAI, GPO/ Letterbook/17, Jan. 1858–Dec. 1878, Letter from W. Crofton to Governor, Mountjoy Prison, 23 April 1858.

75 See Cox and Marland, *Disorder Contained*, *op. cit.* (note 4), 66–8 for the marks system.

76 Sixth RDCPI 1859, 1860 (2655), 22.

77 Edward Balme Wheatley, *Observations on the Treatment of Convicts in Ireland with Some Remarks on the Same in England by Four Visiting Justices of the West Riding Prison at Wakefield* (London: Simpkin, Marshall, and Co., 1862).

78 W.L. Clay, *Our Convict Systems* (Cambridge: Macmillan and Co., 1862), 43.

79
*Ibid.*, 44.

80
*Ibid.*, 45–6, 41.

81
*Ibid.*, 46.

82 Wheatley, *Observations on the Treatment of Convicts in Ireland*, *op. cit.* (note 77), 7–8.

83
*Report and Observations on the Discipline and Management of Convict Prisons, by the Late Major-General Sir Joshua Jebb* (London: Hatchard and Co., 1863).

84
*Ibid.*, 55.

85 T.J. Hatton and J.G. Williamson, *The Age of Mass Migration: Causes and Economic Impact* (New York and Oxford: Oxford University Press, 1998), 20.

86
*Report and Observations on the Discipline and Management*, *op. cit.* (note 83), 855–6.

87 Twelfth RDCPI 1865, 1866 (3745), 17.

88
*Ibid.*, 18–19; NAI, GPO/ Letterbook /7/ Jan. 1865–Dec. 1867, Letter from Director Murray to Lord Mayo, 3 October 1867. See special issue of *Journal of the History of Medicine and Allied Sciences*, 73, 2 (2018) for articles on food as medicine.

89 For details, see Cox and Marland, *Disorder Contained*, *op. cit.* (note 4), 80, n.14.

90 Quote taken from Edward Gibson, ‘Penal Servitude and Tickets of Leave’, *Journal of the Statistical and Social Inquiry Society of Ireland*, 3 (April 1863), 332–43, p.335.

91 See, for example, Wiener, *Reconstructing the Criminal, op. cit.* (note 10); Cox and Marland, *Disorder Contained*, *op. cit.* (note 4), ch. 3; Forsythe, *The Reform of Prisoners*, *op. cit.* (note 7), chs 6 and 8.

92 Priestley, *Victorian Prison Lives*, *op. cit.* (note 10), 51.

93 Reports of Committee to Inquire into Dietaries of Convict Prisons (1864) [467], 3.

94 See Priestley, *Victorian Prison Lives*, *op. cit.* (note 10), ch. 7 for prisoners’ responses to Graham’s dietary scales.

95 Jabez Spencer Balfour, *My Prison Life* (London: Chapman and Hall, 1907), 75.

96 One Who Has Tried Them, *Her Majesty’s Prisons: Their Effects and Defects*, Vol. 2 (London: Sampson Low, Marsten, Searle, & Rivington, 1881), 90.

97 Priestley, *Victorian Prison Lives*, *op. cit.* (note 10), 148.

98 Sim, *Medical Power in Prisons, op. cit.* (note 10), 34–5; McConville, *English Local Prisons*, *op. cit.* (note 3), 118–21, 304.

99 Sim, *Medical Power in Prisons, op. cit.* (note 10), 36.

100 Hardy, ‘Development of the Prison Medical Service’, *op. cit.* (note 8), 68, 70.

101 Martin Wiener, ‘The Health of Prisoners and the Two Faces of Benthamism’, in Creese, Bynum, and Bearn (eds), *The Health of Prisoners*, 44–58, 53.

102 Quote in ibid., 55.

103 Report of the Commissioners Appointed to Inquire into the Operation of the Acts (16 & 17 Vict. c.99 and 20 & 21 Vict. c.3.) relating to Transportation and Penal Servitude, 1863 (3190, 3190–1), 42.

104

Mary Carpenter , Reformatory Prison Discipline as Developed by the Rt. Hon. Sir Walter Crofton in the Irish Convict Prisons (London: Longmans, Green, Reader, and Dyer, 1872), 15 ; Report of the Commissioners on Transportation and Penal Servitude, Evidence of Sir Walter Crofton, *op.cit.* (note 103), 255.

105 Report of the Committee on Dietaries in County and Borough Gaols, Ireland 1867–68 [3981], 29.

106
*Ibid.*, 23, 40.

107 Report of the Committee on Dietaries of the Prisons of England and Wales (1878) [C.95], 5; McConville, *English Local Prisons*, *op. cit.* (note 3), 305.

108 Royal Commission into Penal Servitude Acts, Minutes of Evidence (Kimberley Commission) (1878–9) [C.2368 C.2368-I C.2368-II], 11–20.

109 Sim, *Medical Power in Prisons, op. cit.* (note 10), 35.

110 McConville, *English Local Prisons*, *op. cit.* (note 3), 304.

111 Report of the Committee on Dietaries, Ireland, *op. cit.* (note 105), 19.

112 Report of the Committee on Dietaries, England and Wales, *op. cit.* (note 107), 29.

113 McConville, *English Local Prisons*, *op. cit.* (note 3), 306.

114 Third Report of the General Prisons Board, Ireland, 1880–81 with appendices, 1881 [3067], 2.

115 Kimberley Commission, *op. cit.* (note 108), xvi, xxiii.

116 NAI, GPB/CORR/1880/15475, Dietary Questions: Reports of Medical Officers; Royal Commission on Prisons in Ireland, Second Report, 1884 [C.4145], 17.

117 Prisons (Ireland). Copies of Rules with Respect to the Diets of Prisoners Confined in Ordinary Prisons, 1878 [C. 314], 5; Report of the Committee on Dietaries, England and Wales, *op. cit.* (note 107), 37; NAI, GBP/CORR/1883/12591, Return of Stimulants Used in Hospital in August 1882, 1 September 1883.

118 Balfour, *My Prison Life, op. cit.* (note 95), 77.

119
*Ibid.*, 113. Prisoners of a higher class, such as ex-businessman and politician Balfour, were more likely to be removed to the infirmary if their health was seen to be at risk.

120 Kimberley Commission, Minutes, Evidence of R.E. Power, *op. cit.* (note 108), 749.

121
*Ibid.*

122 ‘One Who Has Tried It, ‘What Prison Life Is Really Like’, *The Windsor Magazine* (2 July 1895), 197–201, p.200.

123
*Ibid.*, 201.

124 NAI, GPB/CORR/1880/15475, Dr Kelly, HMP Drogheda to the General Prisons Board, 8 July 1880.

125
*Ibid.*, Dr H. Minchin, HMP Grangegorman to the General Prisons Board, 23 June 1880.

126
*Ibid.*, Dr R. Kinkead, HMP Galway to the General Prisons Board, 5 June 1880.

127
*Ibid.*, Dr E. Thompson, HMP Omagh to the General Prisons Board, 12 June 1880.

128
*Ibid.*, Dr J. Moore, HMP Belfast to the General Prisons Board, n.d.

129
*Ibid.*, Dr Kinkead, HMP Galway to the General Prisons Board, 5 June 1880; *ibid.*, Dr D. Hadden, HMP Wexford to the General Prisons Board, 7 June 1880.

130 NAI, GPO/ XB/3/1846-49. Convict Prisons Minute book, 8 February 1847.

131 Birmingham Central Library, Wolfson Centre, AX Pamphlets, Vol. 16, 64872: Joseph Allday *, True Account of the Proceedings Leading to, and a Full & Authentic Report of, The Searching Inquiry, by Her Majesty’s Commissioners, into the Horrible System of Discipline Practised at the Borough Gaol of Birmingham* (Birmingham: John Tonks, 1853), 63–9 .

132
*Ibid.*, 63; Cox and Marland, *Disorder Contained*, *op. cit.* (note 4), 49–51.

133
*The Ulster Gazette*, 30 August 1879, 1.

134
*Ibid.*

135
*Ibid.*, 21 June 1879, 3.

136
*The Western Daily Press,* 11 July 1879, 3.

137 Thos. Wemyss Bogg, ‘Disease and Prison Diet’, *Lancet*, 114, 2919 (11 July 1879), 225.

138 Thirteenth RDCPI, 1866 with appendix, 1867 (3805), 20, 21; Cox and Marland, *Disorder Contained*, *op. cit.* (note 4), 112.

139 Allday*, True Account*, *op.cit.* (note 131), 67.

140 See entries in NAI, GPO/PN/4 and GPO/PN/5: Philipstown Character Books, 1847–1862.

141 Kimberley Commission, Minutes, Evidence of Capt. J.P.A. Lewis, *op. cit.* (note 108), 96.

142 Report of the Committee on Dietaries, England and Wales, *op. cit.* (note 107), 28–31, p.31.

143
*Ibid.*, 27.

144
*Ibid.*, 5.

145 Smith and Milner, ‘Report on the Action of Prison Diet and Discipline’, *op. cit.* (note 51), 51.

146

Michael Davitt and Carla King , Jottings in Solitary (Dublin: University College Dublin Press, 2003), xiii; 4 Hansard, LV, col. 1183, 28 March 1898.

